# Protocol for Disome-seq to identify transcriptome-wide ribosome collisions in yeast cells

**DOI:** 10.1016/j.xpro.2025.104047

**Published:** 2025-08-26

**Authors:** Pedro H. Ayres-Galhardo, James Marks, Sezen Meydan

**Affiliations:** 1 Department of Biochemistry, Vanderbilt University, Nashville, TN 37232, USA; 2 Vanderbilt Brain Institute, Vanderbilt University, Nashville, TN 37235, USA; 3 Vanderbilt-Ingram Cancer Center, Vanderbilt University Medical Center, Nashville, TN 37232, USA

**Keywords:** Cell Biology, Genomics, Sequencing, Model Organisms, Molecular Biology, Gene Expression

## Abstract

When translation elongation is hindered, stalled ribosomes can collide with trailing ribosomes and form ribosome collision complexes also known as disomes. Disomes are sensed in the cell to trigger signaling events, and therefore it is important to determine their frequency and distribution across transcripts. Here, we provide the protocol for Disome-seq in *Saccharomyces cerevisiae* (yeast) cells to enable transcriptome-wide detection of disomes. We describe the steps for yeast growth and isolation of disomes as well as sequencing library preparation.

For complete details on the use and execution of the protocol, please refer to Meydan et al.^1^ and Meydan et al.^2^

## Highlights

• Procedures for efficient lysis of yeast cells by filtering and cryo-milling
• Isolation of monosomes and disomes by sucrose gradient fractionation
• Enrichment of disome footprints during ribosome profiling library preparation

## Abstract (editor highlights)

Publisher’s note: Undertaking any experimental protocol requires adherence to local institutional guidelines for laboratory safety and ethics.

## Graphical abstract

**Figure:**
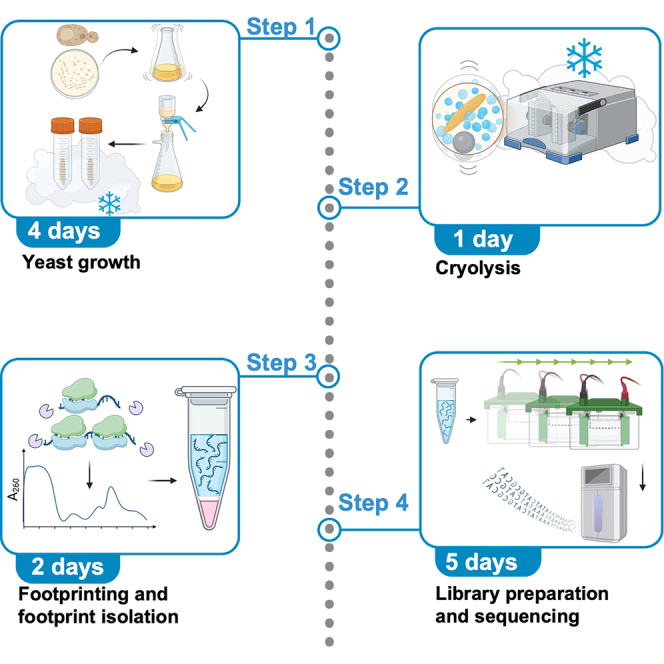

## Before you begin

The protocol below outlines the Disome-seq approach to detect the sites of ribosome collisions in *S. cerevisiae* (yeast) cells. We included the steps for growth and lysis of yeast cells, isolation of footprints protected by disomes, and subsequent library preparation.^3^ We successfully used this technique in different *S. cerevisiae* strains to determine basal distribution of ribosome collisions,^1^ as well as to detect how ribosome collisions are affected by cellular stress conditions such as loss of ribosome collision sensors,^1^ amino acid deficiency,^1^ and oxidative stress.^2^

**CRITICAL:** Since RNA samples are prone to degradation, it is imperative to maintain equipment and reagents free of contaminants, particularly those with nuclease activity.

**CRITICAL:** Reusable components of the protocol such as those used in yeast lysis should be thoroughly cleaned with purified water after each use.

## Key resources table

**Table:** 

REAGENT or RESOURCE	SOURCE	IDENTIFIER
**Chemicals, peptides, and recombinant proteins**		

Tris-HCl, pH 8.0 (1 M)	Thermo Fisher Scientific	AM9855G
Tris-HCl, pH 7.5 (1 M)	Thermo Fisher Scientific	15-567-027
2 M KCl	Thermo Fisher Scientific	AM9640G
1 M MgCl_2_	Thermo Fisher Scientific	AM9530G
Triton X-100	Sigma-Aldrich	93443-100ML
Cycloheximide	Sigma-Aldrich	C1988-1G
Water	Thermo Fisher Scientific	AM9930
Dithiothreitol (DTT)	Sigma-Aldrich	3860-5GM
Sucrose	Sigma-Aldrich	84097-1KG
RNase I (100 U/μL)	Thermo Fisher Scientific	AM2294
Acid phenol solution, saturated with 0.01 M citrate buffer, pH 4.3 ± 0.2	Sigma-Aldrich	P4682
Sodium dodecyl sulfate (SDS) solution 20%	Sigma-Aldrich	05030
Chloroform	Sigma-Aldrich	C2432
Isopropanol	Sigma-Aldrich	I9516
3 M NaOAc (sodium acetate)	Thermo Fisher Scientific	AM9740
Molecular biology grade ethanol	Sigma-Aldrich	E7023-500ML
0.5 M EDTA	Thermo Fisher Scientific	AM9260G
10% SDS	Sigma-Aldrich	71736-100ML
Bromophenol blue sodium salt	Sigma-Aldrich	2830-25GM
Xylene cyanol FF	Sigma-Aldrich	X4126-10G
Urea	Sigma-Aldrich	51456-500G
Ficoll 400	Sigma-Aldrich	F2637-10G
10X TBE	Thermo Fisher Scientific	AM9865
Small RNA marker (RNA ladder)	Abnova	R0007
SYBR Gold	Thermo Fisher Scientific	S11494
15% Criterion TBE-urea polyacrylamide gel, 12 well	Bio-Rad	3450091
Glycoblue	Thermo Fisher Scientific	AM9516
Adenylation mix	NEB	E2610
NEBuffer 1	NEB	B7001S
Oligo Clean & Concentrator	Zymo	D4061
T4 PNK	NEB	M0201L
T4 RNA ligase 2 truncated K227Q	NEB	M0351L
5′ deadenylase	NEB	M0331S
RecJ exonuclease	Biosearch Technologies	RJ411250
Saccharomyces cerevisiae Ribo-Seq riboPOOL	Galen Molecular	dp-K012-49
SUPERase-In	Thermo Fisher Scientific	AM2696
5 M NaCl	Thermo Fisher Scientific	AM9760G
10% Criterion TBE-urea polyacrylamide gel, 18 well	Bio-Rad	3450089
Superscript III reverse transcriptase	Invitrogen	56575
dNTP mix	NEB	N0447S
10 M NaOH	Sigma-Aldrich	72068-100ML
O’RangeRuler 10 bp DNA ladder	Thermo Fisher Scientific	SM1313
CircLigase ssDNA ligase	Epicentre	CL4115K
29:1 40% acrylamide	Bio-Rad	1610146
N,N,N′,N′-tetramethylethylenediamine (TEMED)	Sigma-Aldrich	T7024-25ML
Ammonium persulfate	Fisher Scientific	BP17925
Glycerol	Sigma-Aldrich	G5516-1L
Phusion polymerase	Thermo Fisher Scientific	F530S
High sensitivity D1000 ScreenTape, part of the TapeStation systems	Agilent	5067-5584
High sensitivity D1000 reagents	Agilent	5067-5585

**Experimental models: Organisms/strains**		

*Saccharomyces cerevisiae* BY4741	Meydan et al., 2020^1^	N/A
*Saccharomyces cerevisiae* SUB280	Meydan et al, 2023^2^	N/A
*Saccharomyces cerevisiae* S288C	Meydan et al, 2023^2^	N/A

**Oligonucleotides**		

See Table S1 for oligonucleotide sequences		

**Other**		

Beckman Coulter DU730 UV-vis spectrophotometer	Beckman Coulter	A23616
NanoDrop One/One^C^ microvolume UV-vis spectrophotometer	Fisher Scientific	ND-ONE-W
DWK Life Sciences Kimble Kontes Ultra-Ware microfiltration assembly with fritted glass support (90 mm)	Fisher Scientific	K953755-0090
DWK Life Sciences Kimble Kontes Ultra-Ware heavy-wall filter flasks	Fisher Scientific	K953760-1002
Whatman nitrocellulose NC45 filter discs 0.45 μm pore size	MiliporeSigma	WHA7184009
Micro spatulas	MiliporeSigma	Z513385
labForce centrifuge tubes in trays, 50 mL (30 X 115 mm), molded with graduations, polypropylene with screw cap	Thomas Scientific	1158R10
Cryomill (2012) 100–240 v 50/60 Hz	Retsch	207490001
5 mL stainless steel cryomill jar	Retsch	014620290
Adapter for 5 mL cryomill jars	Retsch	027060304
Seal for 5 mL cryomill jars	Retsch	031110262
Stainless steel 7 mm grinding ball	Retsch	53680030
Cryomill hose/vent with adapter	Retsch	058710001
Screw cap micro tube, 1.5 ml, sterile	Sarstedt	72.692.005
Seal-Rite 1.5 mL microcentrifuge tubes, sterile	USA Scientific	1615-5510
SW 41 Ti swinging-bucket rotor	Beckman Coulter	331362
13.2 mL, Thinwall polypropylene tubes, 14 × 89 mm	Beckman Coulter	331372
Beckman L8-55 UltraCentrifuge	Beckman Coulter	BECK-0L8-0055
Gradient Station base unit	Biocomp	153
SW41 tube mount and accessories	Biocomp	151-114A
9/16" (14 mm) MagnaBase holder and marker block	Biocomp	105-914A-IR
Triax flow cell for dual 260 and/or 280 nm scans	Biocomp	FC-2-UV-260/280
Gilson Fraction FC-203B collector mfg# 171011G 100-240v	Biocomp	151-203
Screw cap micro tube, 2 ml, sterile	Sarstedt	72.693.005
Eppendorf ThermoMixer C	Fisher Scientific	05412503
Eppendorf ThermoMixer C SmartBlock 1.5 mL	Fisher Scientific	05412505
Scientific Industries Vortex-Genie 2 Mixer, variable speed, 120 V	Fisher Scientific	50728002
Disrupter Genie 120 V analog	Fisher Scientific	50728078
4150 TapeStation System	Agilent	G2992AA

## Materials and equipment

### Preparation of lysis buffer

**Table:** Lysis buffer

Reagent	Final concentration	Amount
Tris-HCl, pH 8.0 (1 M)	20 mM	200 μL
KCl (2 M)	140 mM	700 μL
MgCl_2_ (1 M)	1.5 mM	15 μL
Triton X-100 (10%)	1%	1 mL
Cycloheximide (10 mg/mL)	0.1 mg/mL	100 μL
RNase-free water	N/A	7.985 mL
**Total**	**N/A**	**10 mL**

**CRITICAL:** Cycloheximide is toxic.

**CRITICAL:** Triton X-100 is an irritant.

### Preparation of frozen lysis buffer droplets

**Timing: 1 h**

***Note:*** Prepare fresh and keep on ice if not freezing.

1. For each sample, prepare a 50 mL conical tube by poking four holes at the bottom of it with a 21 gauge needle.
2. Fill a liquid nitrogen-safe bucket with liquid nitrogen.
3. Place the perforated conical tubes in a tube holder so that the tubes are filled with the liquid nitrogen.

***Note:*** The liquid nitrogen should reach between 5-10 mL level on the tube.

4. Dropwise add 750 μL of lysis buffer to the conical tube to obtain frozen droplets inside the tube.

***Note:*** Raise your pipette above the container between drops to prevent the buffer from freezing inside the pipette tip.

***Note:*** Do not pipette the buffer into the same spot each time. This would create an amorphous clump instead of the desired single droplets.

5. Drain the liquid nitrogen from the holes of the conical tubes into the bucket by lifting the tubes, then cap them and place them in −80°C freezer.

**Pause Point:** Frozen droplets of lysis buffer can be kept in −80°C freezer indefinitely.

### Setting up the cryomill program

6. Connect the CryoMill to a full liquid nitrogen tank.
7. Turn on the machine and set up the following program: auto pre-cooling, then 9 cycles of 1 minute at 30 Hz and 2 minutes at 5 Hz rest.

**Table:** 

Step	Hz	Time	Cycles
Pre-cooling	5	30 s	1
Grinding	30	60 s	9
Rest	5	120 s

8. Make sure the sealing plug on the right of the chamber and lock nuts on the left are tightened.

**CRITICAL:** If the sealing plug and lock nuts are not tightened properly, this can lead to injuries and malfunctioning of the equipment.

9. Start the program and do a test run: if there is no error message, end it after 1 or 2 cycles.

**Table:** Sucrose gradient buffer (10X)

Reagent	Final concentration	Amount
Tris-HCl, pH 8.0 (1 M)	200 mM	4 mL
KCl (2 M)	1.5 M	15 mL
MgCl_2_ (1 M)	50 mM	1 mL
DTT (1 M)	5 mM	100 μL
**Total**	**N/A**	**20.1 mL**

**CRITICAL:** DTT is an irritant.

***Note:*** Make a fresh 1 M DTT solution from powder.

***Note:*** Prepare the sucrose gradient buffer fresh and keep on ice.

**Table:** 60% Sucrose solution

Reagent	Final concentration	Amount
Sucrose	60%	150 g
RNase-free water	N/A	100 mL
**Total**	**N/A**	**250 mL**

***Note:*** First, solubilize sucrose with 100 mL of water in a 250-mL beaker on a hot plate with stirring, and then transfer to a measuring cylinder or volumetric flask to complete the volume to 250 mL with RNase-free water.

***Note:*** Filter-sterilize the 60% sucrose solution using 0.22 μM filters and store at 4°C.

**Table:** 10% Sucrose solution in gradient buffer

Reagent	Final concentration	Amount
60% Sucrose	10%	6.6 mL
10X Gradient buffer	1x	4 mL
RNase-free water	N/A	29.4
**Total**	**N/A**	**40 mL**

**Table:** 50% Sucrose solution in gradient buffer

Reagent	Final concentration	Amount
60% Sucrose	50%	33.3 mL
10X Gradient buffer	1x	4 mL
RNase-free water	N/A	2.7
**Total**	**N/A**	**40 mL**

**Table:** 80% Ethanol

Reagent	Final concentration	Amount
Molecular biology grade ethanol (100%)	80%	80 mL
RNase-free water	N/A	20 mL
**Total**	**N/A**	**100 mL**

**CRITICAL:** Ethanol is volatile and flammable.

***Note:*** Keep this at −20°C and use it for washing pellets after nucleic acid extractions.

**Table:** 10 mM Tris pH 8.0

Reagent	Final concentration	Amount
1 M Tris-HCl pH 8.0	10 mM	100 μL
RNase-free water	N/A	9.9 mL
**Total**	**N/A**	**10 mL**

***Note:*** Keep this at room temperature and use it for resuspending pellets after nucleic acid extractions.

**Table:** RNA Gel extraction buffer

Reagent	Final concentration	Amount
3 M NaOAc	0.3 M	75 μL
0.5 M EDTA	1 mM	1.5 μL
10% SDS	0.25%	18.75 μL
RNase-free water	N/A	654.75 μL
**Total**	**N/A**	**750 μL**

**CRITICAL:** SDS is an irritant.

**CRITICAL:** EDTA poses health hazards.

***Note:*** Prepare fresh and keep at room temperature.

**Table:** 0.1% Bromophenol blue sodium salt solution

Reagent	Final concentration	Amount
Bromophenol blue sodium salt	0.1%	10 mg
RNase-free water	N/A	10 mL
**Total**	**N/A**	**10 mL**

***Note:*** Prepare ahead of time and keep at 4°C.

**Table:** 0.1% Xylene cyanol FF solution

Reagent	Final concentration	Amount
Xylene cyanol FF	0.1%	10 mg
RNase-free water	N/A	10 mL
**Total**	**N/A**	**10 mL**

***Note:*** Prepare ahead of time and keep at 4°C.

**Table:** 1% Bromophenol blue sodium salt solution

Reagent	Final concentration	Amount
Bromophenol blue sodium salt	1%	100 mg
RNase-free water	N/A	10 mL
**Total**	**N/A**	**10 mL**

***Note:*** Prepare ahead of time and keep at 4°C.

**Table:** 1% Xylene cyanol FF solution

Reagent	Final concentration	Amount
Xylene cyanol FF	1%	100 mg
RNase-free water	N/A	10 mL
**Total**	**N/A**	**10 mL**

***Note:*** Prepare ahead of time and keep at 4°C.

**Table:** 2X TBU solution

Reagent	Final concentration	Amount
Urea	7 M	4.2 g
Ficoll 400	12% w/v	1.2 g
10X TBE buffer	1X	1 mL
**Total**	**N/A**	**10 mL**

***Note:*** Dissolve Urea and Ficoll 400 in TBE by mixing with a magnetic stirrer on a heated plate, and then add RNase-free water to complete the volume to 10 mL.

***Note:*** Prepare ahead of time and keep 1 mL aliquots at 4°C.

**Table:** SYBR Gold staining buffer

Reagent	Final concentration	Amount
SYBR Gold Nucleic Acid Gel Stain (10,000X)	1X	10 μL
10X TBE	1X	10 mL
RNase-free water	**N/A**	90 mL
**Total**	**N/A**	**100 mL**

***Note:*** Make 10 μL aliquots of 10,000x SYBR Gold stock solution to minimize freeze-thaw and light exposure.

**Table:** DNA Gel extraction buffer

Reagent	Final concentration	Amount
5 M NaCl	0.3 M	45 μL
0.5 M EDTA	1 mM	1.5 μL
1 M Tris pH 8.0	10 mM	7.5 μL
RNase-free water	N/A	696 μL
**Total**	**N/A**	**750 μL**

**CRITICAL:** EDTA poses health hazards.

***Note:*** Prepare ahead of time and keep at 4°C for up to two weeks.

**Table:** 10X DNA Gel loading buffer

Reagent	Final concentration	Amount
100% Glycerol	50%	5 mL
1% Bromophenol blue sodium salt	0.05%	0.5 mL
1% Xylene cyanol FF	0.05%	0.5 mL
RNase-free water	N/A	4 mL
**Total**	**N/A**	**10 mL**

***Note:*** Prepare ahead of time and keep at 4°C.

## Step-by-step method details

### Growth and filtering of yeast cells

**Timing: 4–5 days**

**Timing: Variable (for step5)**

This step describes the growth of yeast cells and filtering the media to obtain yeast cell scrapes.

Day 1

1. Retrieve a yeast glycerol stock of the strain of interest and streak it on an appropriate agar plate.
2. Allow the yeast to grow for 48 hours.

***Note:*** The streaked yeast plate can be covered with a parafilm and be kept at 4°C for a month.

Day 3

3. Using the streaked agar plate, start 5 mL of overnight cultures of yeast in the appropriate selection media or Yeast Extract Peptone Dextrose (YPD) in a shaker at 30°C and 200 rpm.
4. Prepare and sterilize 750 mL media in a 2 L flask for each yeast culture for the growth that will take place next day.

Day 4

5. Prepare the yeast culture.
  a. Measure the optical density at 600 nm (OD600) of 1:10 diluted overnight culture.
    ***Note:*** Spectrophotometers differ on reported OD600 value: generate a growth curve prior to this experiment and determine if your spectrophotometer reports values other than OD600 of 0.5 as the mid-log phase.
  b. Based on the measurement obtained in Step 5a, multiplied by 10, inoculate the 750 mL media with overnight yeast culture to reach a final OD600 of ∼0.02.
  c. Grow the yeast at 30°C and periodically check the OD600 of the culture until it reaches mid-log phase (OD600 of 0.5–0.6).
    ***Note:*** For slow growing yeast strains, first grow a 25 mL of yeast culture until they reach mid-log phase at day 4, then inoculate the 750 mL media with this mid-log phase culture starting at an OD600 of ∼0.005, grow it overnight, and then harvest at day 5. Note that this extra step would increase the number of days for growth to 5 days.
    ***Note:*** For treatment of yeast cells, such as hydrogen peroxide, start the treatment once the culture reaches mid-log phase.
6. While the culture is growing, prepare two 50 mL conical tubes per each culture for filtration by piercing three holes at the bottom of the conical tubes with a 21 gauge needle.

***Note:*** Keep these pierced tubes on dry ice or at −80°C freezer.

7. Once the cultures are getting close to the mid-log phase, prepare the filtration assembly.
  a. Wet a Whatman nitrocellulose filter disc with purified water and place it on top of the glass vacuum filter so that the filter disc and glass vacuum filter circle line up.
  b. Insert vacuum filter inside the filter flask.
  c. Carefully place the funnel on top of the vacuum filter-filter flask assembly and use the aluminum clamp to support the connection between vacuum filter and the funnel.
    ***Note:*** Ensure that the entire assembly is lined up.
  d. Connect the filter assembly to a vacuum line.
    ***Note:*** See Figure 1 for an example assembly.
    **Figure 1:**
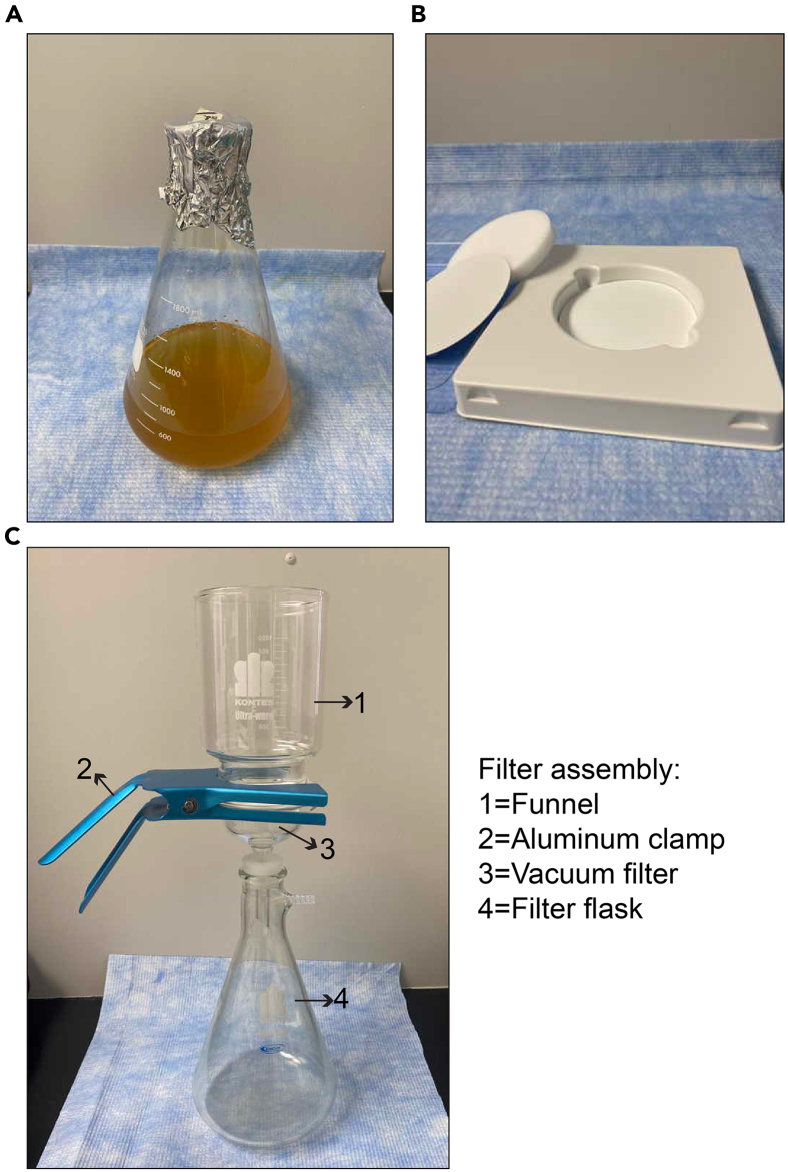
Parts and assembly of yeast filtration apparatus (A) 750 mL mid-log phase yeast culture in a 2 L flask. (B) Whatman nitrocellulose filter disc used in the filter assembly. (C) Filter assembly for filtering the yeast culture.
8. Prepare the liquid nitrogen bucket with pierced tubes.
  a. Fill a liquid nitrogen-safe bucket with liquid nitrogen.
  b. Take off the caps of the conical tubes from Step 6.
  c. Place the tubes in a tube holder inside the liquid nitrogen bucket so the tubes are filled with liquid nitrogen.
9. Pour 750 mL of mid-log phase yeast culture into the funnel and immediately turn on the vacuum.
10. Once all the culture media is filtered through and no more media is left in the funnel, turn off the vacuum, disconnect the vacuum tubing, and promptly remove the funnel by disassembling the aluminum clamp.
11. Scrape the cells with metal spatulas.
  a. Using the flat ends of two clean spatulas, one in each hand, carefully but quickly scrape all the yeast on the filter disc.
    ***Note:*** You should obtain two scrapes per each culture.
  b. Place the cell scrapes into the two tubes inside the liquid nitrogen-filled bucket and wait until you hear a sizzling sound, indicating that they are being frozen.
12. Once the scrapes are frozen and solid, use the rounded end of a clean, dry spatula to dislodge the scrapes from the spatulas submerged in liquid nitrogen, close the lids of the conical tubes and place them in −80°C freezer.
13. Clean all the parts of the assembly thoroughly with purified water, discard the filter disc.
14. Prepare the filtration assembly as described in Step 7 but omit the filter disc. Pour 1 L of purified water into the funnel and turn on the vacuum.

***Note:*** This additional step is strongly recommended to keep the filtration assembly clean.

**Pause Point:** Cell scrapes in conical tubes can be stored indefinitely in −80°C freezer.

### Cryolysis of yeast cell scrapes

**Timing: 1 day**

### This step describes the preparation of yeast lysates

**Figure 2:**
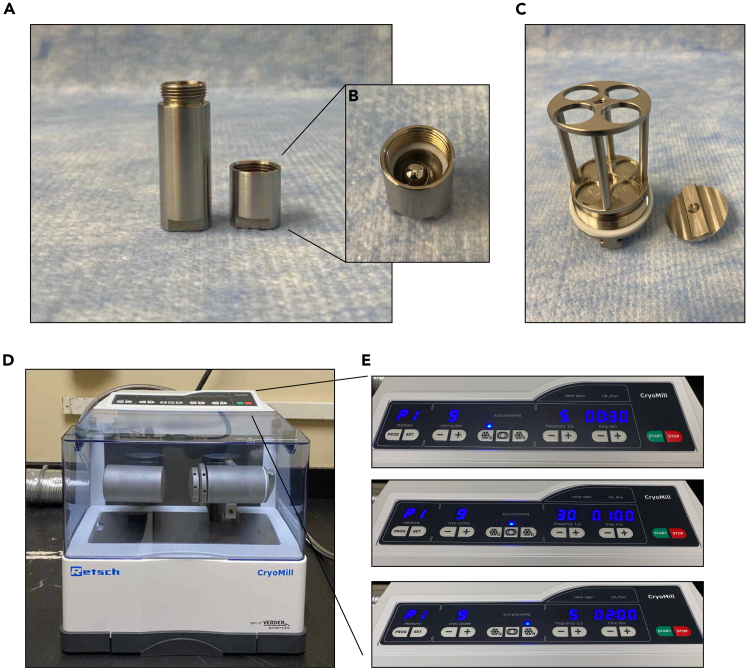
Cryolysis parts and settings (A) Capsule and capsule lid. (B) Capsule lid and metal ball. (C) Canister. (D) CryoMill. Pre-cooling (E), Grinding (F), and Rest (G) settings at the CryoMill screen.
18. Load the grinding jar into the CryoMill.
  a. Unscrew the sealing plug on the right side of the chamber by using the opening aid that is provided with the CryoMill equipment.
    ***Note:*** These opening aids should be stored on two sides of the machine on top of the support slots.
  b. Loosen the lock nut and the screw with the locking pin that is provided with the equipment.
    ***Note:*** Make sure the sealing rings (grinding jar, grinding jar support, cooling jacket) are correctly inserted and undamaged.
  c. Slide the grinding jar into the cooling jacket and completely screw the grinding jar in by using the opening aid.
  d. Using the locking pin, secure the grinding jar by first tightening the screw and then tighten the lock nut firmly against the cooling jacket.
19. Start the liquid nitrogen flow to the machine.
20. Run the CryoMill program of auto pre-cooling followed by 9 cycles of 1 minute at 30 Hz and 2 minutes at 5 Hz rest, saved as in the “Before you start” section.

***Note:*** After 9 cycles have passed, come back and manually re-start the cycle (18 cycles total).

15. Prepare the capsules and the grinding jar on dry ice.
  a. Make sure to number the capsules (1 to 4) both on top of the capsule lids and at the bottom of the 5 mL capsules with a marker.
  b. For each sample, place a capsule and its corresponding lid on dry ice.
    ***Note:*** The top of the lid should be facing the dry ice.
  c. Place one 7 mm metal ball in the capsule and another 7 mm ball inside the lid.
16. Place the frozen droplets of lysis buffer and cell scrapes into the capsules.
  a. Once the capsules, lids, and metal balls cool down on dry ice for 10 minutes, take out and place on dry ice one of the two conical tubes containing a cell scrape described in “growth and filtering of yeast cells” section and a conical tube with frozen droplets of lysis buffer described in “preparation of lysis buffer” section.
  b. Using clean, dry tweezers, transfer the frozen cell scrapes to a capsule.
    ***Note:*** Dip the tweezers briefly in liquid nitrogen to avoid any potential melting of the scrape during transfer to capsule.
  c. Transfer all the frozen lysis buffer droplets (750 μL) by carefully pouring them into the capsule.
    ***Note:*** If the droplets are stuck at the bottom of the conical tube, gently tap the tube to release the frozen droplets.
  d. Drop the metal ball in the lid into the bottom capsule containing frozen droplets, cell scrape, and the other metal ball, and screw the lid on tightly.
    ***Note:*** All the components of the lysate need to be fully frozen for an efficient lysis, so move the droplets and cell scrapes to the capsules as quickly as possible, and always keep them on dry ice.
    **CRITICAL:** The metal capsules on dry ice are very cold, so use a well-fitting, thin (for easy handling) cryoprotection glove for holding them while screwing the lid.
17. Load the capsules into the grinding jars.
  ***Note:*** Lids of the capsules should be touching the sealing lid of the grinding jar.
  a. Dip the entire canister in liquid nitrogen for 1 minute.
  b. Keep the assembly on dry ice until you load into the CryoMill equipment.
    ***Note:*** See Figure 2 as a reference for the parts and CryoMill settings.
    **CRITICAL:** If you are using less than 4 capsules, make sure they are balanced at the opposite side of the grinding jar.

21. Once the milling is finished, remove the grinding jar from the CryoMill cooling jacket and turn off the machine.
  a. Unscrew the lock nut and the screw by using the locking pin.
  b. Open the grinding jar by using the opening aid, and place the grinding jar on dry ice.
  c. Immediately place the sealing plug back.
  d. Turn off the liquid nitrogen flow and switch off the machine.
  e. Place paper towels under the hood of the CryoMill to avoid condensation inside the chamber.
22. Remove the lysed cells out of the grinding jar.
  a. Open the grinding jar by using two opening aids.
  b. Take out one capsule at a time, leave the other capsules on dry ice.
  c. Unscrew the capsule lid, check it for powdered lysate and place it upside down on top of a clean napkin.
  d. Drop the contents of the capsule by inverting it into a conical tube.
  e. By using a clean and dry set of tweezers, remove the 2 metal balls and wash them with purified water for future use.
  f. By using the rounded end of a clean and dry spatula, scoop the remaining powdered lysate from the capsule and capsule lid into the conical tube.
  g. Melt the powdered lysate in the conical tube on ice.
  h. Wash the capsules and capsule lids thoroughly with purified water.
23. Once melted, clarify samples by centrifugation.
  a. In a prechilled centrifuge, spin at 3,000 x g, 4°C for 5 minutes to pellet the cell debris.
    ***Note:*** Use a fixed-angle rotor so the pellet is at the wall of the conical tube.
  b. Transfer the supernatant into a 1.5 mL microcentrifuge tube and discard the pellet.
    ***Note:*** When transferring, avoid pipetting the white lipid layer.
  c. Spin down the lysate at 21,000 x g, 4°C for 10 minutes.
  d. Transfer the supernatant into a 1.5 mL screwcap freezer tube and discard the pellet.
    ***Note:*** When transferring, avoid pipetting the white lipid layer.
  e. Take 1 μL of the supernatant and dilute it in 249 μL of water.
  f. Measure the absorbance at 260 nm (A260).
  g. Multiply the A260 reading by the dilution factor (250) and the volume in mL to calculate the “A260xVolume” of the lysate.
    ***Note:*** For example, a typical A260 reading of 1:250 diluted lysate of 600 μL (0.6 mL) volume would be ∼1.5. Therefore, total A260 in this case would be 1.5x250x0.6=225.
  h. Aliquot the lysate into tubes containing ∼50 A260xVolume to prevent frequent freeze and thaw.
    ***Note:*** For the example in Step 23g, this sample could be aliquoted into 4 tubes.
  i. Flash freeze the sample in liquid nitrogen.
    **Pause Point:** Cell lysates can be stored indefinitely in −80°C freezer.

### Sucrose gradient fractionation of monosomes and disomes

**Timing: 1 day**

This step describes the footprinting and isolation of monosomes and disomes by sucrose gradient fractionation.

24. Set the ultracentrifuge to 40,000 rpm, 4°C, 3 hours and let it cool down under vacuum.

***Note:*** Ensure that the swinging buckets and rotor are placed at 4°C.

25. Thaw cell lysates at room temperature, aliquot the lysate for the intended A260xVolume obtained in the “Cryolysis of Yeast Cell Scrapes” section, and equal volume of fresh lysis buffer to dilute the lysate.

***Note:*** A260xVolume of 45 is recommended for this protocol.

26. Add 11.25 μL RNase I to the lysate and mix well by pipetting.
27. Incubate for 1 hour with gentle agitation on a thermomixer (700 rpm, 22°C), and then adjust the total volume of lysate to 800 μL with fresh lysis buffer.
28. Prepare 10% and 50% sucrose solutions in gradient buffer during the incubation and make 10-50% gradient.
  a. Mark the outside of the propylene centrifuge tubes by using the high side of marker block.
  b. Fill propylene centrifuge tubes with 6 mL of 10% sucrose solution in gradient buffer by using a pipettor.
  c. Attach the canula to a luer lock 10 mL syringe and slowly dispense 50% sucrose solution in gradient buffer from the bottom of the centrifuge tube.
    ***Note:*** You should see two layers form upon dispensing 50% sucrose solution.
    ***Note:*** The canula itself will affect the height of the interface: as you dispense sucrose solution, slowly lift cannula so that it stays 2-3 cm below the interface.
  d. Remove the canula from the tube and set aside.
  e. Wipe off the remaining sucrose from the canula before filling the next tube.
  f. Carefully put on short rubber caps.
    ***Note:*** When inserting the rubber caps, begin with the side opposite of the hole first, allowing excess sucrose to escape the tube, and remove excess sucrose from the top of the cap.
  g. Run the Biocomp gradient making program:
    i. Select the “GMST” option.
    ii. Using the provided balance, level the magnetic base.
    iii. Select the “GRAD” option.
    iv. If preparing a gradient for the first time, select the “LIST” option under the gradient menu and set your rotor model; SW41 in this case.
    v. Then, set the gradient concentration range to “10-50%”. A shortcut can be taken in future runs by selecting “LAST” under the gradient menu.
    vi. Select “RUN”.
  h. Remove the rubber caps carefully starting from the hole.
  i. Carefully pipette out 1 mL from the top of each tube and discard.
    ***Note:*** 10–50% sucrose gradients can also be prepared the night before and be kept at 4°C overnight.
29. Slowly load the sample dropwise to the inner wall above the sucrose gradient, careful to minimize disruption to the top of the gradient.
30. Insert the centrifuge tubes into the swinging-bucket rotor buckets and make sure that the opposing buckets weigh the same by using an analytical balance.

***Note:*** For example, first weigh one bucket placed in a tube rack, tare, then weigh the pairing bucket (Bucket number 1 vs 4, 2 vs 5, and 3 vs 6): If it displays −0.10 g, it means you need to add ∼100 μL of 1X gradient buffer. If it is 0.10 g, then it means you need to add ∼100 μL of 1X gradient buffer to the former bucket.

**Figure 3:**
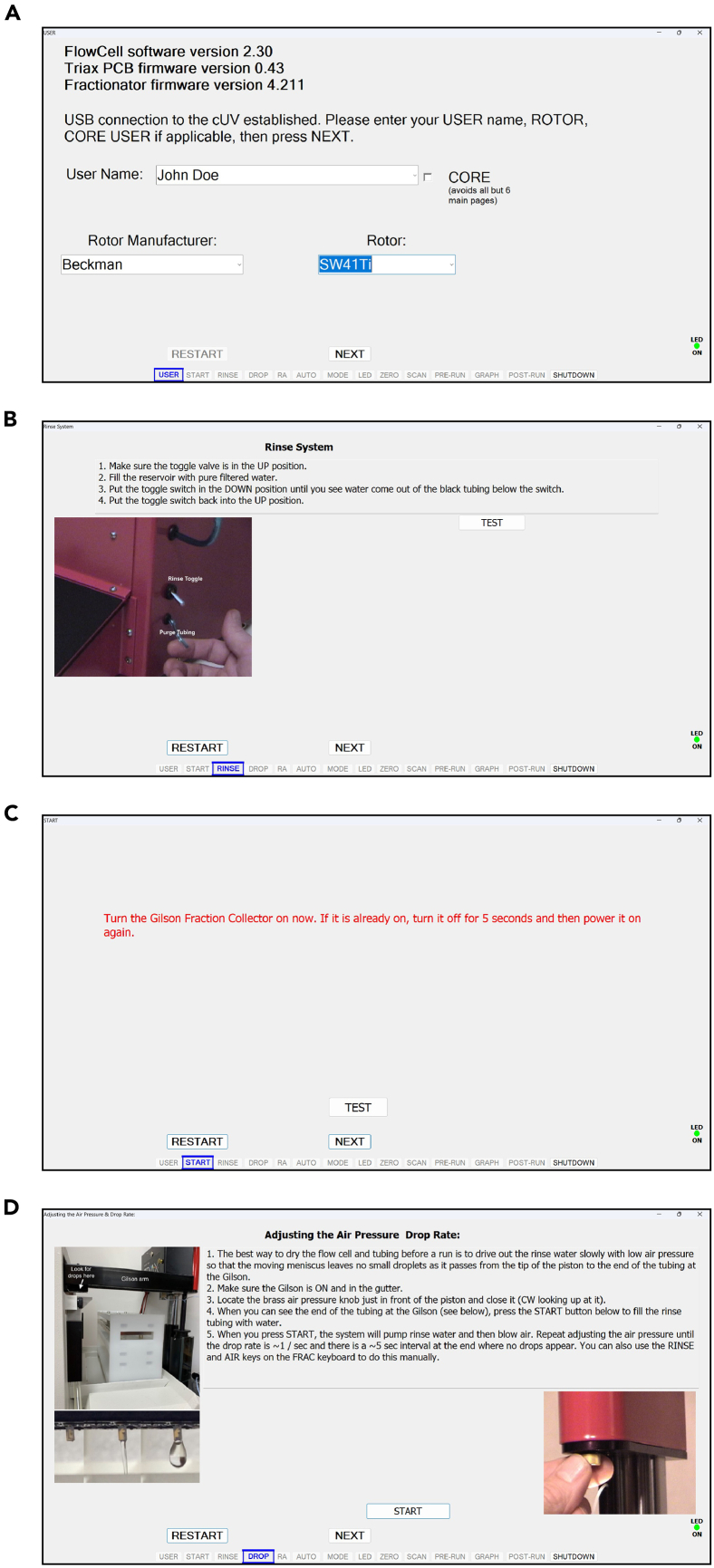
Biocomp fractionator user interface snapshots demonstrating the set-up for the username, computer connection, and drop rate (A) When typing a username for the first time, the software will create a folder under that name in the local system to store run information. (B) The test button can be used to verify the connection between the computer and the fraction collector. (C) Power on Gilson Fraction Collector and validate its connection. (D) The drop rate can be adjusted by turning the brass knob. The drop rate calibration does not need to be repeated after initial use if the brass knob remains undisturbed.

**Figure 4:**
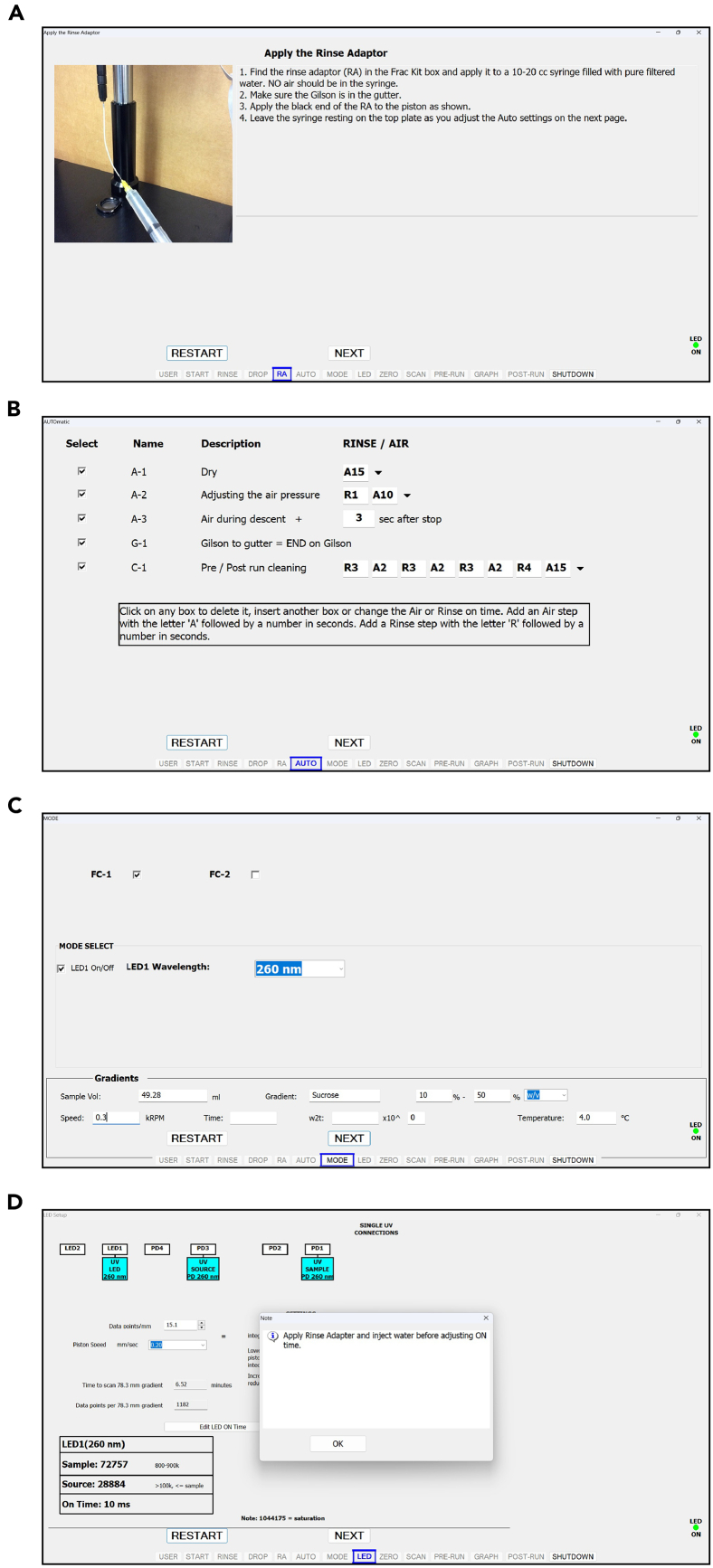
Biocomp fractionator user interface snapshots of adaptor rinsing, auto and mode settings and LED instructions (A) Flush the nozzle using the rinse adapter. (B) The recommended automated rinsing and air pumping steps. (C) Select 260 nm absorption using a single LED. (D) Follow the instructions in the pop-up page on the “LED” page.

**Figure 5:**
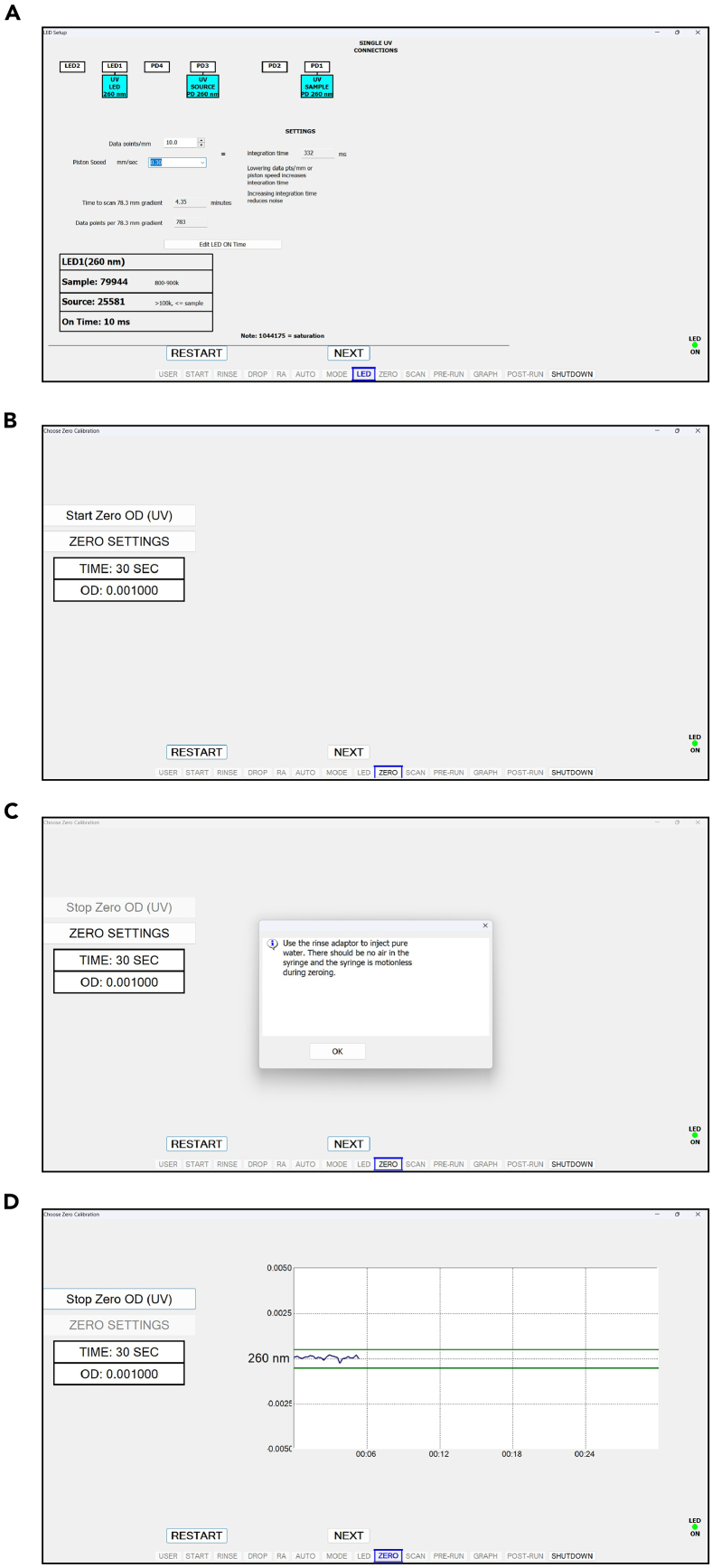
Biocomp fractionator user interface snapshots for LED settings and 260 nm baseline absorbance calibration (A) Select the options above for the “LED” page. (B) Select the “Start Zero OD (UV)” option above. (C) Follow the instructions in the pop-up page. (D) UV blanking over 30 seconds.

31. Hang the buckets on the rotor with the matching numbers and ensure that they are safely inserted.
32. Place the rotor in the ultracentrifuge, and spin the rotor at 40,000 rpm, 4°C, 3 hours.
33. Once the centrifugation is completed, fractionate the samples using the Gradient Station according to instructions on Figures 3, 4, 5, and 6.

**Figure 6:**
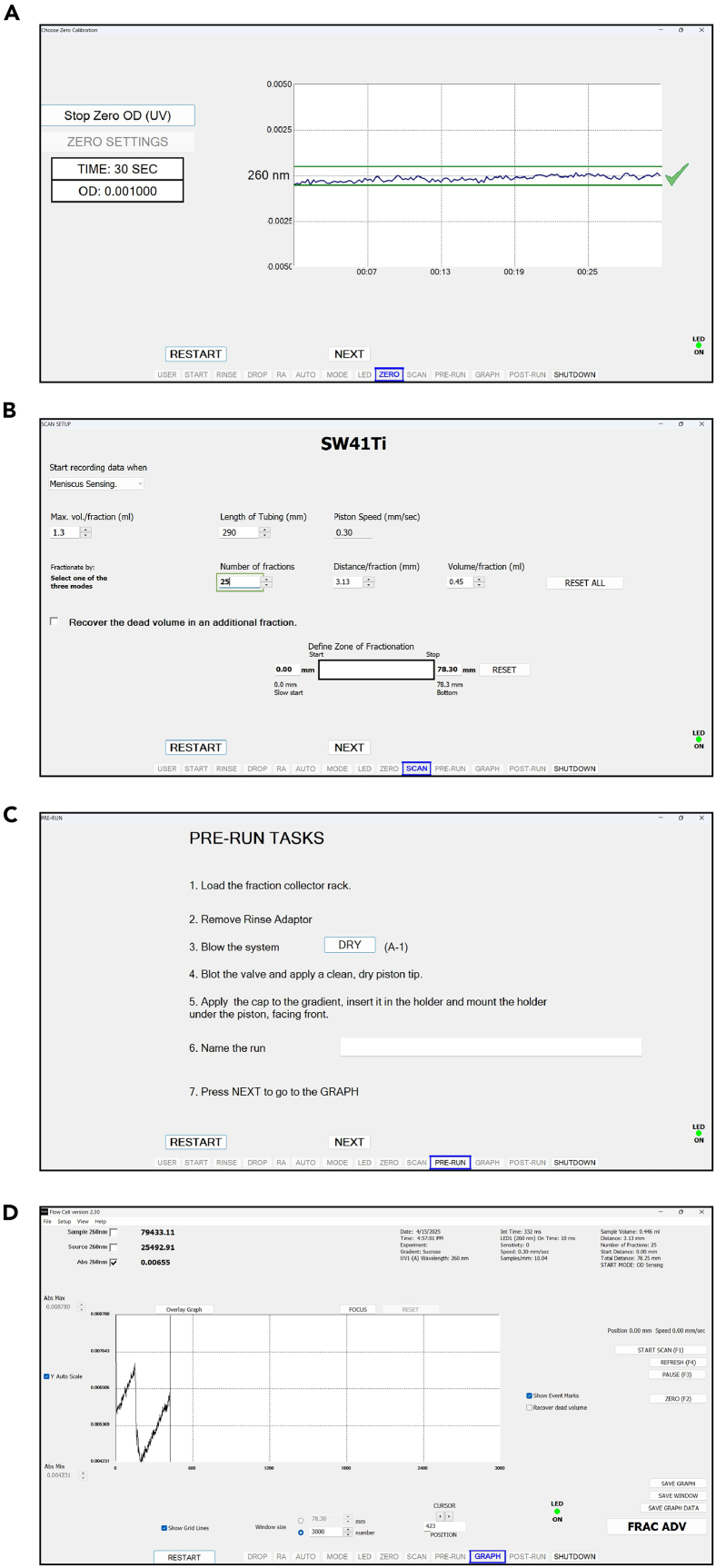
Biocomp fractionator user interface snapshots for completed 260 nm absorbance calibration, fractionation settings and initiation of fractionation (A) The read should stay within the bounds marked in green. A check mark will appear at the end of a successful read. (B) Select the options above for the “SCAN” page. (C) Follow the steps above to prepare the system for your samples. (D) Select the “START SCAN” option to start fractionation.
34. Collect the fractions containing monosome and disome peaks, see an example gradient in Figure 7.

**Figure 7:**
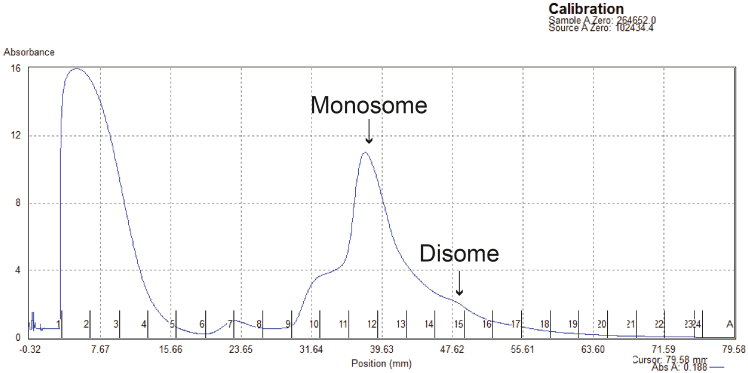
Example of sucrose gradient performed in lysates obtained from wild-type *S. cerevisiae* (BY4741) In this example, sucrose gradient is 10%–50%. Monosome and disome peaks used for preparing Ribo-seq and Disome-seq libraries, respectively, are indicated with arrows.

***Note:*** If the disome is spread across two fractions, use a pipette and tube inversion to combine the different sucrose gradient fractions into a homogenous mixture to prevent uneven sampling.

35. Aliquot the combined fractions into 1 mL samples and flash freeze them with liquid nitrogen.

**Pause Point:** The sucrose fractions can be stored in −80°C indefinitely.

### Extraction of footprints from sucrose gradient fractions

**Timing: 3 h**

### This step describes the purification of monosome and disome footprints

**CRITICAL:** Phenol and chloroform are particularly hazardous substances, wear gloves, lab coat, and safety glasses during these experiments.

**CRITICAL:** The steps 36–45 need to be performed in a chemical fume hood.

36. Set up tubes for acid phenol/chloroform extraction.
  a. Prepare and label two 1.5 mL screw cap tubes per each sample (that has 1 mL sucrose gradient fraction) and fill them with 375 μL of acid phenol.
  b. Heat the tubes with acid phenol at 65°C for 10 minutes in a thermomixer.
  c. Prepare and label one 2 mL screw cap tube per each sample, fill it with 700 μL of acid phenol, and leave at room temperature.
  d. Prepare and label one 1.5 mL screw cap tube per each sample, fill it with 600 μL of chloroform, and leave at room temperature.
37. Add 57.1 μL of 20% SDS per 1 mL of the collected sucrose fraction and heat the tubes at 65°C to dissolve SDS while inverting the tube several times to ensure mixing.
38. Once the fraction + SDS becomes clear, add 500 μL of it to each of the two pre-heated phenol tubes prepared in Step 36a-b.
  a. Incubate for 2 min at 65°C, and vortex.
  b. Incubate for 2 min at 65°C, and vortex.
  c. Incubate for 1 min at 65°C.
39. Place the samples on ice and incubate for 5 minutes.
40. Spin for 2 minutes at full speed (21,000 x g) at room temperature.
41. Pull the aqueous layer.
  a. Pipette out the **bottom** layer in each tube into the 2 mL screw cap tube that contains 700 μL of room temperature acid phenol (prepared in Step 36c) by combining two tubes per each sample into one tube.
    ***Note:*** Because of high concentration of sucrose, the aqueous layer is at the bottom instead of top.
  b. Vortex for 5 minutes by using a disruptor that can hold multiple tubes.
42. Spin for 2 minutes at full speed (21,000 x g) at room temperature.
43. Pull the aqueous layer.
  a. Pipette out the **bottom** layer in each tube into the 1.5 mL screw cap tube that contains 600 μL of room temperature chloroform (prepared in Step 36d).
    ***Note:*** Because of high concentration of sucrose, the aqueous layer is at the bottom instead of top.
  b. Vortex for 30 seconds.
    ***Note:*** If the sample looks cloudy, place them back in the 65°C thermomixer for 1 minute before removing the aqueous layer.
44. Spin for 2 minutes at full speed (21,000 x g) at room temperature.
45. Pull the **top** aqueous layer (∼1 mL) into 15 mL tubes on ice.
46. Prepare the precipitation reaction and place the tubes in −80°C for 30 minutes.
  a. During this incubation, pre-cool a microcentrifuge to 4°C and prepare 3 x 1.5 mL microcentrifuge tubes per each sample.

**Table:** Precipitation reaction

Reagent	Final concentration	Amount
Sample	N/A	1 mL
RNase-free water	N/A	1 mL
3 M NaOAc	0.15 M	222 μL
Isopropanol	N/A	2.222 mL
**Total**	**N/A**	**4.444 mL**

***Note:*** You can also leave the samples at −80°C for overnight precipitation.

**CRITICAL:** Isopropanol is volatile and flammable.

47. Thaw the precipitation reaction tubes at room temperature and separate each into 3 x 1.5 mL microcentrifuge tubes.
48. Spin for 30 minutes at full speed (21,000 x g) at 4°C.
49. Remove the supernatant and wash the white pellet containing RNA with 750 μL 80% cold ethanol.
50. Spin for 5 minutes at full speed (21,000 x g) at 4°C.
51. Remove the supernatant with a P1000 pipettor, quick spin the samples on a benchtop mini centrifuge, then remove the rest of the supernatant with a P200 pipettor.
52. Air dry the pellets for 10 minutes at room temperature by leaving the microcentrifuge tube lids open, and visually check to ensure that the pellet is fully dry.
53. Resuspend the pellets with 10 mM Tris pH 8.0 and combine the three tubes into one.
  a. For disome samples, resuspend one of the three tubes with 7 μL 10 mM Tris pH 8.0 and then use this resuspension to resuspend the pellets from two other tubes (total volume is 7 μL).
  b. For monosome samples, resuspend each of the three tubes with 7 μL 10 mM Tris pH 8.0 and then combine them all into a single tube (total volume is 21 μL).
54. Dilute the samples 1:20 (0.5 μL of the sample + 9.5 μL water) and measure A260 and A260/A280 values.

**Pause Point:** These RNA samples can be kept at −80°C indefinitely.

### Size selection of footprints

**Timing: 3, 16–20, and 2 h (1.5 days total)**

### This step describes the size selection of ribosome footprints

55. Determine the volume that corresponds to a maximum of 5 μg of RNA from Step 54 and aliquot it into a 1.5 mL microcentrifuge tube.

***Note:*** If the sample volume is smaller than 10 μL, dilute the sample with 10 mM Tris pH 8.0 to 10 μL.

***Note:*** Libraries can be successfully prepared with samples that have as low as 500 ng input material.

56. Prepare the size markers.

**Table:** Monosome size markers

Reagent	Final concentration	Amount
25 nucleotide RNA size marker (100 μM)	2.5 μM	1 μL
34 nucleotide RNA size marker (100 μM)	2.5 μM	1 μL
10 mM Tris pH 8.0	4.5 μM	18 μL
2X TBU	1X	20 μL
**Total**	**N/A**	40 μL

**Table:** Disome size markers

Reagent	Final concentration	Amount
54 nucleotide RNA size marker (100 μM)	2.5 μM	1 μL
68 nucleotide RNA size marker (100 μM)	2.5 μM	1 μL
10 mM Tris pH 8.0	4.5 μM	18 μL
2X TBU	1X	20 μL
**Total**	**N/A**	40 μL

57. Prepare the RNA ladder.

**Table:** RNA ladder dilution

Reagent	Final concentration	Amount
RNA ladder	N/A	2 μL
2X TBU	1.6X	8 μL
**Total**	**N/A**	10 μL

58. Prepare the color dye.

**Table:** Color dye

Reagent	Final concentration	Amount
Bromophenol blue sodium salt solution (0.1%)	0.025%	2.5 μL
Xylene cyanol FF solution (0.1%)	0.025%	2.5 μL
2X TBU	1X	5 μL
**Total**	**N/A**	10 μL

59. Incubate samples, markers and RNA ladder for 2 minutes at 80°C, and then place immediately on ice.
60. Prepare and load the size selection gel.
  a. Place a 15% TBE-Urea gel in a tank filled with 1X TBE.
    **CRITICAL:** Polyacrylamide gels can contain acrylamide, which is neurotoxic.
  b. Clean the wells of the gel by pipetting up and down to each well with a P1000 pipette.
  c. Load the entire sample, 7 μL of size markers at each end of the gel, 2.5 μL of RNA ladder at each end of the gel and 5 μL of the color dye at one of the empty wells.
  d. Leave empty wells between samples to avoid cross-contamination.
  e. Load all empty wells with 5 μL 2X TBU.
    ***Note:*** See Figure 8 for an example size selection gel.
    **Figure 8:**
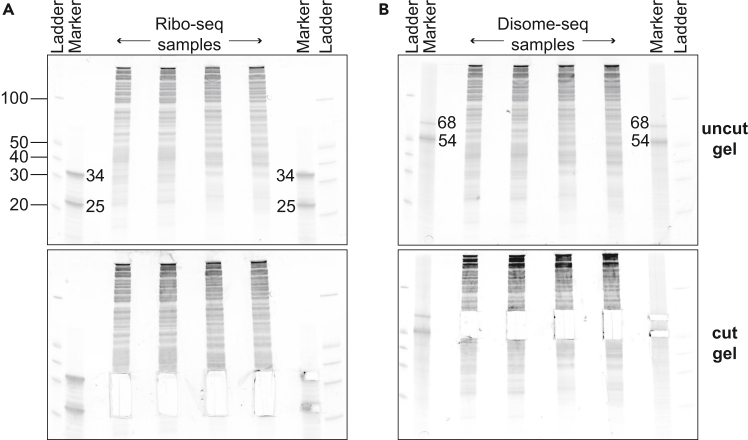
Example of size selection gels Size selection gels of Ribo-seq (A) and Disome-seq (B) samples. RNA ladder sizes are shown with black lines. Size markers for Ribo-seq (25-34mers) and Disome-seq (54-68mers) are indicated on each gel image. The gel pieces encompassing the area of the size marker at other lanes are cut for extraction (lower panel).
61. Run the gel at 7 or 15 Watts constant for 1 or 2 gels, respectively, until front dye (bromophenol blue) reaches the bottom of the gel, while the trailing dye (xylene cyanol) is still at the top.
62. Remove the gel out of its cassette and stain it with 1X SYBR Gold solution by shaking it for 5 minutes while covered in foil.
63. Transfer the gel onto a transparent plastic laser printer sheet and cover it with a Saran Wrap.
64. Image the gel using an imager system that has a SYBR Gold setting and a blue light transilluminator for placing the gel, and save the uncut gel image.

***Note:*** Blue light transilluminator must be used to avoid potential ultraviolet light damage of the RNA.

65. Place the gel on a blue light transilluminator and cut the region of the sample in line with the outer edges of the corresponding size markers by using a razor blade.

***Note:*** See Figure 8 for an example cut gel.

66. Place the cut gel piece into a 1.5 mL microcentrifuge tube.
67. Once the sample lanes are cut, then cut the size markers as well and place them into a 1.5 mL microcentrifuge tube (25+34 and 54+68 nucleotide size markers combined as monosome and disome footprint marker, respectively).
68. Add 500 μL RNA Gel Extraction Buffer to each tube and freeze them in −80°C for 30 minutes.

**Pause Point:** The buffer-frozen gel pieces can be kept at −80°C indefinitely.

69. Place the tubes with buffer-frozen gel pieces in a thermomixer that is set to 22°C, 700 rpm overnight.
70. Next day, briefly spin down samples by using a benchtop mini centrifuge and transfer the supernatant to a fresh 1.5 mL microcentrifuge tube.

***Note:*** Avoid getting any gel pieces into the fresh tube and repeat the spin if you observe any small gel pieces.

71. Start precipitation of RNA.
  a. Add 2 μL glycoblue and 500 μL isopropanol to each tube.
  b. Mix well by inverting the tube 8 times.
  c. Place samples on ice and incubate for 1 hour.
72. Spin for 30 minutes at full speed (21,000 x g) at 4°C.
73. Remove the supernatant and wash the blue pellet with 750 μL 80% cold ethanol.
74. Spin for 3 minutes at full speed (21,000 x g) at 4°C.
75. Remove the supernatant with a P1000 pipettor, quick spin the samples on a benchtop mini centrifuge, then remove the rest of the supernatant with a P200 pipettor.
76. Air dry the pellets for 10 minutes at room temperature by leaving the microcentrifuge tube lids open, and visually check to ensure that the pellet is fully dry.
77. Resuspend the pellets with 3.5 μL 10 mM Tris pH 8.0.

**Pause Point:** These RNA samples can be kept at −80°C indefinitely.

### Ligation of barcoded linkers to footprints

**Timing: 1 day**

This step describes the preparation of pre-adenylated linkers, dephosphorylation of footprints, and ligation of linkers to dephosphorylated footprints.

78. Prepare the adenylation reactions in a 200 μL PCR tube.

**Table:** Adenylation reaction for each linker

Reagent	Final concentration	Amount
10X 5′ DNA adenylation reaction buffer	1X	2 μL
1 mM ATP	100 μM	2 μL
100 μM barcoded linker (NI-810, NI-811, NI-812, NI-813, NI-814 or NI-815	6 μM	1.2 μL
50 μM Mth RNA ligase	5 μM	2 μL
RNase-free water	N/A	12.8 μL
**Total**	**N/A**	20 μL

79. Place the PCR tubes in a thermocycler and incubate them at 65°C for 1 hour, followed by 85°C for 5 minutes.
80. Transfer the PCR tube contents into a 1.5 mL microcentrifuge tube and add 30 μL RNase-free water to complete the volume to 50 μL.
81. Clean up the adenylated linkers by using Oligo Clean & Concentrator Kit.
  a. Mix 100 μL Oligo Binding Buffer with the sample.
  b. Add 400 μL ethanol and mix well.
  c. Load samples onto the Zymo Spin-Column placed inside a collection tube.
  d. Centrifuge the column for 30 s at 12,000 x g and discard the flowthrough.
  e. Wash the column with 750 μl DNA Wash Buffer, centrifuge for 30 s at 12,000 x g, and discard the flow-through.
  f. Centrifuge again for 1 min at maximum speed to remove any residual wash buffer.
  g. Transfer the Zymo-Spin Column into a 1.5 mL microcentrifuge tube and add 6 μl RNase-free water.
  h. Centrifuge for 30 s at 12,000 x g to elute the pre-adenylated linker.

**Pause Point:** The pre-adenylated linkers can be kept at −20°C as 0.7 μL aliquots to avoid multiple freeze-thaw.

82. Prepare the dephosphorylation reaction.

**Table:** Dephosphorylation reaction

Reagent	Final concentration	Amount
Size selected RNA Sample/size marker	N/A	3.5 μL
10X T4 PNK Buffer	1X	0.5 μL
SUPERase-In 20 U/μL	2 U/μL	0.5 μL
T4 PNK 10 U/μL	1 U/μL	0.5 μL
**Total**	**N/A**	5 μL

83. Mix the tubes by gently flicking and collect the contents at the bottom by briefly centrifuging, then incubate the dephosphorylation reaction mix at 37°C for 1 hour.
84. Prepare the ligation reaction.

**Table:** Ligation reaction

Reagent	Final concentration	Amount
Dephosphorylated RNA Sample/size marker	N/A	5 μL
10X T4 RNA Ligase Buffer	0.5X	0.5 μL
20 μM pre-adenylated linker barcode	1 μM	0.5 μL
50% PEG-8000	17.5%	3.5 μL
T4 RNA Ligase truncated K227Q 200 U/μL	10 U/μL	0.5 μL
**Total**	**N/A**	10 μL

85. Incubate the ligation reaction mix at 22°C for 3 hours.
86. Prepare the diluted 5′ deadenylase by scaling up the recipe below at least 5 times to ensure reliable pipetting.

**Table:** Dilution of 5′ deadenylase

Reagent	Final concentration	Amount
NEBuffer 1	N/A	0.05 μL
5′ deadenylase 50 U/μL	10 U/μL	0.1 μL
RNase-free water	N/A	0.35 μL
**Total**	**N/A**	0.5 μL

87. To each sample or size marker, add 0.5 μL of diluted 5′ deadenylase and 0.5 μL of RecJ exonuclease, mix well and incubate at 30°C for 45 minutes.
88. Clean up the samples and size markers using Oligo Clean & Concentrator Kit.
  ***Note:*** At this step, you can pool the samples that have different linker barcodes.
  a. If pooling the samples, calculate the total volume of the pool and complete the volume to 50 μL with water.
    ***Note:*** For example, each sample/size marker volume after dephosphorylation and linker ligation is 11 μL: if 6 samples are being pooled, then the total volume would be 66 μL.
    ***Note:*** For size markers, 39 μL water should be added.
  b. For pooled samples, prepare a microcentrifuge tube with oligo binding buffer 2 times the total pool volume.
  c. Pool the samples in the Oligo Binding Buffer.
    ***Note:*** The denaturing environment of the oligo binding buffer can potentially prevent residual ligase activity and cross-sample ligation.
    ***Note:*** For size markers, mix them with 100 μL Oligo Binding Buffer.
  d. Add ethanol equal to 8 times the total sample pool/marker volume and load the samples into the Zymo Spin-Column placed inside a collection tube.
  e. Centrifuge the column for 30 s at 12,000 x g and discard the flowthrough.
  f. Wash the column with 750 μl DNA Wash Buffer, centrifuge for 30 s at 12,000 x g, and discard the flow-through.
  g. Centrifuge again for 1 min at maximum speed to remove any residual wash buffer.
  h. Transfer the Zymo-Spin Column into a 1.5 mL microcentrifuge tube and add 10 μL RNase-free water.
  i. Centrifuge for 30 s at 12,000 x g to elute the linker-ligated footprints.
    ***Note:*** At this point, we highly recommend the ribosomal RNA (rRNA) depletion by using commercial kits such as siTools riboPOOLs kit. Oligo Clean & Concentrator kit should be used for cleaning up the rRNA-depleted sample, to be eluted with 10 μL RNase-free water.

### Reverse transcription and cDNA selection

**Timing: 3, 16–20, and 2 h (1.5 days total)**

This step describes reverse transcription of linker-ligated footprints and isolation of the resulting cDNA.

89. Add 2 μL of 1.25 μM NI-802 reverse transcription primer to the samples and markers, mix well.
90. Incubate at 65°C for 5 minutes, then immediately place on ice.
91. Prepare reverse transcription reaction master mix (scale up as needed).

**Table:** Reverse transcription master mix for one sample

Reagent	Final concentration	Amount
5X First Strand Buffer	1X	4 μL
10 mM dNTP mix	1.5 mM	1 μL
0.1 M DTT	5 mM	1 μL
SUPERase-In 20 U/μL	1 U/μL	1 μL
**Total**	**N/A**	7 μL

**CRITICAL:** DTT is an irritant.

92. Add 7 μL of the reverse transcription reaction mix to each sample or size marker.
93. Add 1 μL Superscript III Reverse Transcriptase and mix well.
94. Incubate at 55°C for 30 minutes.
95. Add 2.2 μL 1 M NaOH to each tube and mix well, and incubate at 70°C for 20 minutes.

**CRITICAL:** NaOH is corrosive.

96. Complete the volume of each sample/marker to 50 μL with RNase-free water and clean up using Oligo Clean & Concentrator Kit as described in Step 88, and elute with 6 μL water.
97. Add 6 μL 2X TBU to each tube and mix well.
98. Denature samples and markers at 80°C for 2 minutes, then immediately place them on ice.
99. Prepare and load the reverse transcription gel.
  a. Place a 10% TBE-Urea gel in a tank filled with 1X TBE.
  b. Clean the wells of the gel by pipetting up and down to each well.
  c. Load the entire sample and size marker, 2.5 μL of O’range Ruler 10 bp ready-to-load DNA ladder, and 5 μL of the color dye at one of the empty wells.
  d. Leave empty wells between samples to avoid cross-contamination.
  e. Load all empty wells with 5 μL 2X TBU.

**Figure 9:**
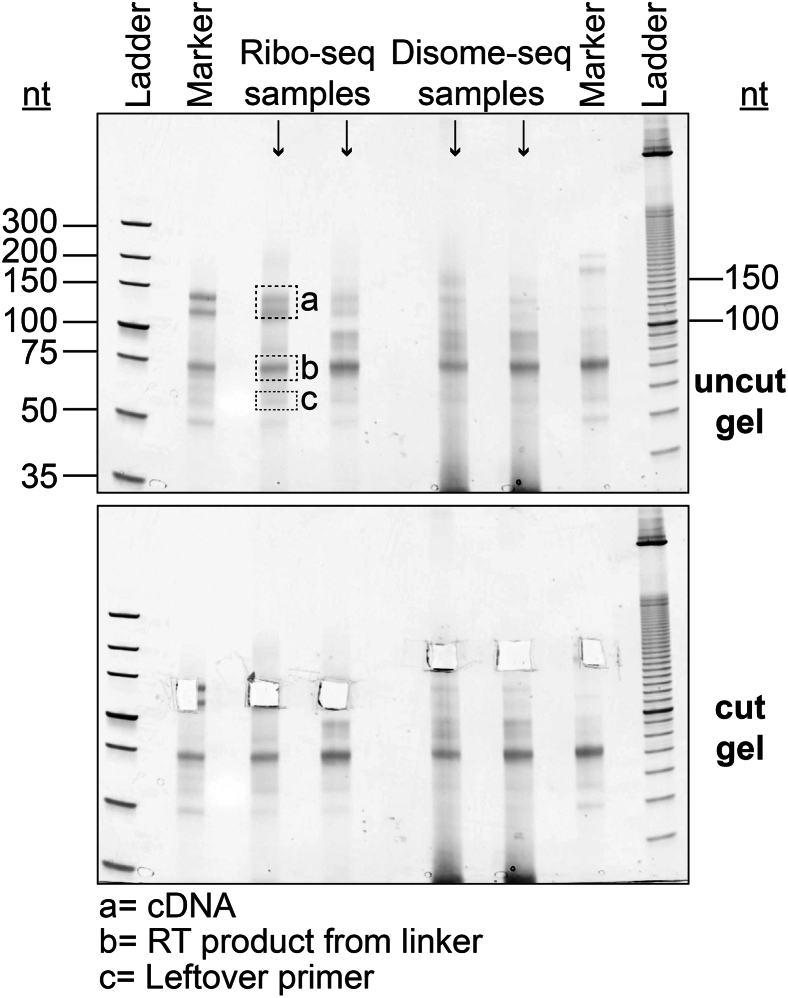
Example of a reverse transcription gel Reverse transcription gel showing Ribo-seq and Disome-seq cDNA products. DNA ladder sizes are indicated with black lines. Reverse transcription products of size markers for Ribo-seq (lane 3) and Disome-seq (lane 13) are indicated on the top gel image. Dashed boxes on the gel (A, B, C) show the cDNA, RT product from linker, and leftover primer as indicated. The gel pieces matching the exact size marker area at other lanes are cut for extraction (lower panel).

***Note:*** See Figure 9 for an example reverse transcription gel.

100. Run the gel at 7 Watts constant until front dye (bromophenol blue) leaves the gel and the trailing dye (xylene cyanol) is about 1 inch off the bottom.
101. Remove the gel out of its cassette and stain it with 1X SYBR Gold solution by shaking it for 5 minutes, covered in foil.
102. Transfer the gel onto a transparent plastic laser printer sheet and cover it with a Saran Wrap.
103. Image the gel using an imager system that has a SYBR Gold setting and a blue light transilluminator for placing the gel, and save the uncut gel image.

***Note:*** Blue light transilluminator must be used to avoid potential ultraviolet light damage of the cDNA.

104. Place the gel on a blue light transilluminator and cut the region of the sample in line with the outer edges of the corresponding size markers by using a razor blade.
105. Place the cut gel piece into a 1.5 mL microcentrifuge tube.
106. Once the sample lanes are cut, then cut the size markers as well and place them into a 1.5 mL microcentrifuge tube.

***Note:*** See Figure 9 for an example cut gel.

107. Add 500 μL DNA Gel Extraction Buffer to each tube and freeze them in −80°C for 30 minutes.

**Pause Point:** The buffer-frozen gel pieces can be kept at −80°C indefinitely.

108. Place the tubes with buffer-frozen gel pieces in a thermomixer that is set to 22°C, 700 rpm overnight.
109. Next day, briefly spin down samples by using a benchtop mini centrifuge and transfer the supernatant to a fresh 1.5 mL microcentrifuge tube.

***Note:*** Avoid getting any gel pieces into the fresh tube and repeat the spin if you observe any small gel pieces.

110. Start precipitation of cDNA.
  a. Add 2 μL glycoblue and 500 μL isopropanol to each tube.
  b. Mix well by inverting the tube 8 times.
  c. Place samples on ice and incubate for 1 hour.
111. Spin for 30 minutes at full speed (21,000 x g) at 4°C.
112. Remove the supernatant and wash the blue pellet with 750 μL 80% cold ethanol.
113. Spin for 3 minutes at full speed (21,000 x g) at 4°C.
114. Remove the supernatant with a P1000 pipettor, quick spin the samples on a benchtop mini centrifuge, and remove the rest of the supernatant with a P200 pipettor.
115. Air dry the pellets for 10 minutes at room temperature by leaving the microcentrifuge tube lids open, and visually check to ensure that the pellet is fully dry.
116. Resuspend the pellets with 15 μL 10 mM Tris pH 8.0.

**Pause Point:** The cDNAs can be kept at −80°C.

### Circularization

**Timing: 1.5 h**

This step describes circularization of the single stranded cDNA.

117. Prepare the circularization reaction mix:

**Table:** Circularization reaction master mix for one sample

Reagent	Final concentration	Amount
cDNA sample	N/A	15 μL
10X CircLigase Buffer	1X	2 μL
1 mM ATP	0.05 mM	1 μL
50 mM MnCl_2_	2.5 mM	1 μL
**Total**	**N/A**	19 μL

118. Add 4 μL of the reaction mix to each sample or marker.
119. Add 1 μL CircLigase ssDNA Ligase and mix well.
120. Incubate at 60°C for 60 minutes, and then at 80°C for 10 minutes to inactivate the CircLigase enzyme.

**Pause Point:** The circularized samples can be kept at −80°C indefinitely.

### Library PCR optimization

**Timing: 3 h**

This step describes the PCR optimization process that will determine the PCR conditions to be used for library preparation PCR.

121. Prepare the PCR master mix:

**Table:** PCR master mix for one sample

Reagent	Final concentration	Amount
5X HF Buffer	1X	6 μL
10 mM dNTP	0.2 mM	0.6 μL
20 μM NI-798 Forward Primer	0.5 μM	0.75 μL
RNase-free water	N/A	21.1 μL
Phusion Polymerase (2 U/μL)	1.2 U/μL	0.3 μL
**Total**	**N/A**	**28.75 μL**

122. Add the circularized template and reverse primer:

**Table:** 

Reagent	Final concentration	Amount
PCR master mix	N/A	28.75 μL
20 μM Reverse Primer	0.5 μM	0.75 μL
Circularized template	N/A	0.5 μL
**Total**	**N/A**	**30 μL**

***Note:*** Size markers should be used along with the samples for ensuring the correct library size on the gel.

***Note:*** Use different reverse primers for sample pools being combined into the same sequencing lane.

123. Aliquot each PCR reaction into 3 PCR tubes, 9 μL each.
124. Using a thermocycler, perform the pilot PCR as described below with three different cycle numbers (7, 10, 13).
125. Pause the PCR at the end of each set number of cycles, remove the corresponding tube, and resume PCR.

**Table:** 

Steps	Temperature	Time	Cycles
Initial Denaturation	98°C	30 sec	1
Denaturation	98°C	10 sec	7, 10, or 13 cycles
Annealing	65°C	10 sec	
Extension	72°C	5 sec

126. Add 1 μL 10X DNA loading buffer to each tube and mix well.
127. Cast 8% native polyacrylamide gel for resolving PCR products.
  a. Prepare the gel mix.

**Table:** Recipe for one 8% native polyacrylamide gel

Reagent	Final concentration	Amount
29:1 40% acrylamide	8%	4 mL
10X TBE	1X	2 mL
RNase-free water	N/A	14 mL
**Total**	**N/A**	**20 mL**

    ***Note:*** If library PCR optimization and preparative PCR will be done in the same day, prepare two gels by scaling up this recipe.
  b. Fully de-gas by using a 50 mL sterilizing filter.
    ***Note:*** Keep the gel mix on ice during this process to avoid rapid polymerization at the next step.
  c. Add 30 μL TEMED and 100 μL 10% APS, mix well by inverting the 50 mL tube multiple times.
    **CRITICAL:** TEMED and APS are skin and respiratory irritants.
  d. Pour the gel mix into an empty cassette and wait for 1 h to fully solidify.
    ***Note:*** Leftover gel mix can be used to gauge solidity of cast gel.
    **CRITICAL:** Polyacrylamide gels can contain acrylamide, which is neurotoxic.
128. Prepare and load the gel.
  a. Place the hand-cast 8% native polyacrylamide gel in a tank filled with 1X TBE.
  b. Load the samples from step 126 along with 2.5 μL of O’range ruler DNA ladder.
129. Run the gel at 15 W or 30 W (for 1 or 2 gels, respectively), until the front dye (bromophenol blue) has reached the bottom of the gel, while the trailing dye (xylene cyanol) is still at the top.
130. Remove the gel out of its cassette and stain it with 1X SYBR Gold solution by shaking it for 5 minutes, covered in foil.
131. Transfer the gel onto a transparent plastic laser printer sheet and cover it with a Saran Wrap.
132. Image the gel using an imager system that has a SYBR Gold setting. Save the gel image.

***Note:*** See Figure 10 for an example library PCR optimization gel.

133. Determine the optimal number of PCR cycles that generate a visible library product on the gel at the correct size, without the presence of higher molecular band weights.
134. Discard the gel and proceed with the preparative, large-scale library PCR.

**Figure 10:**
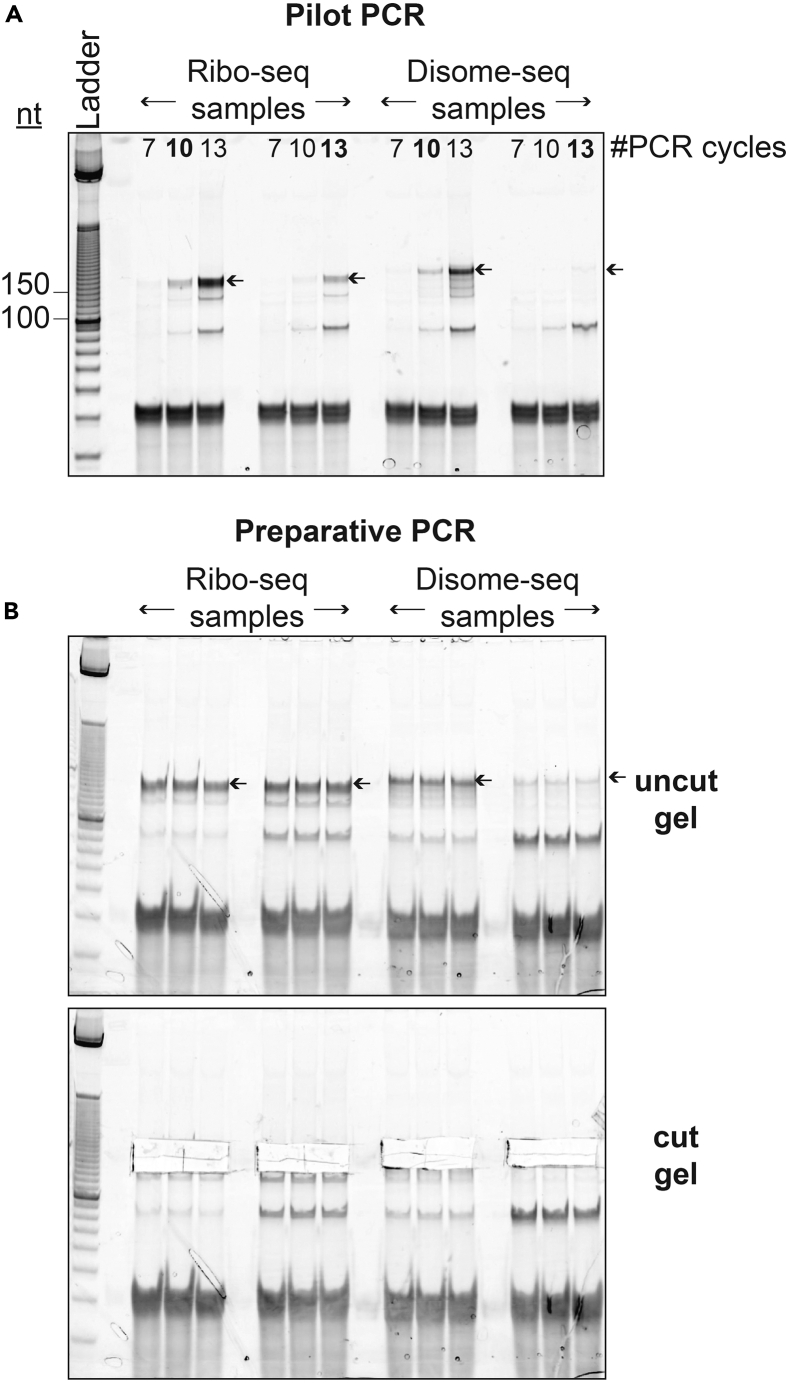
Example of PCR gels (A) Ribo-seq and Disome-seq pilot PCR gel for library optimization. DNA ladder sizes are indicated with black lines. Number of PCR cycles (7, 10, 13) are indicated on top of each PCR lane. The library products are shown with black arrows. The number of PCR cycles selected for preparative PCR, which result in a visible library on the gel while avoiding the high molecular weight bands, is bolted for each sample. (B) Ribo-seq and Disome-seq preparative PCR products. The library products are shown with black arrows. The library product excluding the thin, lower molecular weight band, was excised from the gel for extraction (cut gel, lower panel).

### Preparative library PCR

**Timing: 3, 16–20, and 2 h (1.5 days total)**

This step describes the preparation of final library PCR based on the conditions optimized from the library PCR optimization step.

135. Prepare the PCR master mix:

**Table:** PCR Master mix for one sample

Reagent	Final concentration	Amount
5X HF Buffer	1X	16 μL
10 mM dNTP	0.2 mM	1.6 μL
20 μM NI-798 Forward Primer	0.5 μM	2 μL
RNase-free water	N/A	53.6 μL
Phusion Polymerase (2 U/μL)	1.2 U/μL	0.8 μL
**Total**	**N/A**	**74 μL**

136. Add the circularized template and reverse primer:

**Table:** 

Reagent	Final concentration	Amount
PCR master mix	N/A	74 μL
20 μM Reverse Primer	0.5 μM	2 μL
Circularized template	N/A	4 μL
**Total**	**N/A**	**80** μL

137. Using a thermocycler, perform the PCR as described below for the predetermined amount of cycles.

**Table:** 

Steps	Temperature	Time	Cycles
Initial Denaturation	98°C	30 sec	1
Denaturation	98°C	10 sec	Optimized number of cycles
Annealing	65°C	10 sec	
Extension	72°C	5 sec	
Final extension	72°C	5 min	1

138. Add 9 μL 10X DNA loading buffer to each tube and mix well.
139. Cast 8% native polyacrylamide gel for resolving PCR products as described in Step 127.
140. Prepare and load the gel.
  a. Place the hand-cast 8% native polyacrylamide gel in a tank filled with 1X TBE.
  b. Load the samples from step 138 along with 2.5 μL of O’range ruler DNA ladder.
141. Run the gel at 7 W until the front dye (bromophenol blue) has reached the bottom of the gel, while the trailing dye (xylene cyanol) is still at the top.
142. Remove the gel out of its cassette and stain it with 1X SYBR Gold solution by shaking it for 5 minutes.
143. Transfer the gel onto a transparent plastic laser printer sheet and cover it with a Saran Wrap.
144. Image the gel using an imager system that has a SYBR Gold setting and a blue light transilluminator for placing the gel. Save the uncut gel image.

***Note:*** Blue light transilluminator has to be used to avoid potential ultraviolet light damage of the DNA.

145. Place the gel on a blue light transilluminator and cut the region of the sample in line with the outer edges of the corresponding size markers by using a razor blade.

***Note:*** See Figure 10 for an example cut gel.

146. Place the cut gel piece into a 1.5 mL microcentrifuge tube.
147. Add 500 μL DNA Gel Extraction Buffer to each tube and freeze them in −80°C for 30 minutes.

**Pause Point:** The buffer-frozen gel pieces can be kept at −80°C indefinitely.

148. Place the tubes with buffer-frozen gel pieces in a thermomixer that is set to 22°C, 700 rpm overnight.
149. Next day, briefly spin down samples by using a benchtop mini centrifuge and transfer the supernatant to a fresh 1.5 mL microcentrifuge tube.

***Note:*** Avoid getting any gel pieces into the fresh tube and repeat the spin if you observe any small gel pieces.

150. Start precipitation of the library DNA.
  a. Add 2 μL glycoblue and 500 μL isopropanol to each tube.
  b. Mix well by inverting the tube 8 times.
  c. Place samples on ice and incubate for 1 hour.
151. Spin for 30 minutes at full speed (21,000 x g) at 4°C.
152. Remove the supernatant and wash the blue pellet with 750 μL 80% cold ethanol.
153. Spin for 3 minutes at full speed (21,000 x g) at 4°C.
154. Remove the supernatant with a P1000 pipettor, quick spin the samples on a benchtop mini centrifuge, and remove the rest of the supernatant with a P200 pipettor.
155. Air dry the pellets for 10 minutes at room temperature by leaving the microcentrifuge tube lids open, and visually check to ensure that the pellet is fully dry.
156. Resuspend the pellets with 6 μL 10 mM Tris pH 8.0.

### Quality control assessment and quantification of the library

**Timing: 1 h**

This step describes the TapeStation process for assessing the quality and quantification of the library.

157. Launch the Agilent TapeStation Controller Software.
158. Load High Sensitivity D1000 ScreenTape device and loading tips into the TapeStation instrument.
159. Prepare your sample for the assay by diluting it 1:10 with water (0.5 μL sample, 4.5 μL water).
160. Keep High Sensitivity D1000 reagents at room temperature for 30 min and vortex them before use.
161. Mix 2 μL High Sensitivity D1000 Sample Buffer and 2 μL High Sensitivity D1000 Ladder.
162. Mix 2 μL High Sensitivity D1000 Sample Buffer and 2 μL of the 1:10 diluted library.
163. Quick spin, then vortex using IKA vortexer and adaptor at 2000 rpm for 1 min.
164. Quick spin to position the sample at the bottom of the tube.
165. Load samples into the TapeStation instrument.
166. Select the required samples on the TapeStation Controller Software.
167. Click Start and specify a filename with which to save your results.

**Figure 11:**
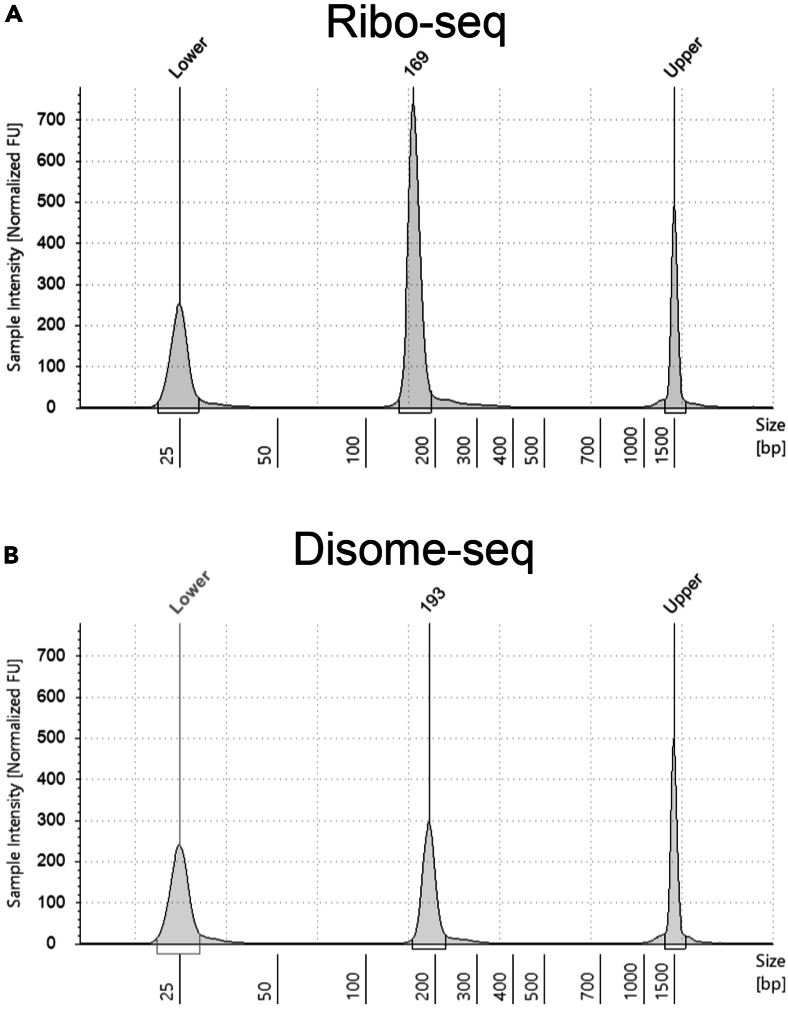
Example of TapeStation analysis of final libraries TapeStation results of a Ribo-seq (A) and Disome-seq (B) library.

***Note:*** See Figure 11 for an example TapeStation output.

***Note:*** The output shows the peaks corresponding to the library: for monosome, the library size should be detected as 169-175, and for disome, the library size should be 190-201 nucleotides.

***Note:*** We observed that the variation reported in the TapeStation library size is not reflected in the footprint sizes. Therefore, a number within the ranges stated above and the presence of a single peak would be acceptable for a library that can be submitted for sequencing.

168. Adjust the concentration of the libraries based on the values obtained in this assay and prepare the samples for sequencing.

***Note:*** The volume and concentration of the library for sequencing should be confirmed with the sequencing facility prior to submission.

***Note:*** Ideal number of sequencing reads per sample should be aimed as 30-50 millions for Ribo-seq and 100-300 millions for Disome-seq, though sequencing data with lower depth can still be informative depending on the research question.

## Expected outcomes

The final output of this protocol is a library of ∼200 nucleotide long fragments (Disome-seq). For Ribo-seq library, the fragment size is ∼170 nucleotides in length. After Illumina sequencing of the library, the reads need to be mapped to yeast genome or transcriptome, and the distribution of disomes should be assessed by bioinformatic analysis.

The Disome-seq data is processed by first demultiplexing the reads in order to separate samples carrying different linkers pooled in Step 88, while trimming the linker sequences from the reads. This step can be done by adapter trimming tools such as Cutadapt.^4^ Then, the reads are mapped to non-coding RNAs, to eliminate the contaminating reads arising from ribosomal RNAs and transfer RNAs. Mapping can be done by tools such as Bowtie.^5^ The reads that are not mapped in this step, devoid of non-coding RNAs, are then de-duplicated by custom scripts to eliminate PCR duplicates resulting from Step 137. Finally, de-duplicated reads are mapped to genome or transcriptome. The quality of the mapped reads is assessed by tools such as FastQC,^6^ to ensure the footprint sizes match the expected disome footprint length and that the data is devoid of overrepresented reads. The expected Disome-seq read size as a result of this protocol is between 57-63 nucleotides. However, some cellular treatment conditions, such as anisomycin, can lead to shorter footprints 51 nucleotides in length.^1^ Disome-seq library preparation should be adjusted to include shorter footprints in this case (40-80 nucleotide size markers instead of 54-68).

Downstream analysis of disome pause scores, their frequency and distribution in each gene are computed by using custom scripts, as exemplified in the repositories provided in Meydan and Guydosh, 2020^1^ and Meydan, Barros et al, 2023.^2^ To assess data quality and validate ribosome positioning, metagene analysis should be performed by averaging read coverage around start and stop codons. The resulting three-nucleotide periodicity confirms characteristic ribosome movement along coding sequences and is the hallmark of a high-quality Disome-seq data.

## Limitations

Although Disome-seq can report on the location of collided ribosomes on transcripts and the sequence features associated with ribosome collisions, it cannot be used to determine the relative level of disomes compared to translating ribosomes in the cell.

## Troubleshooting

### Problem 1

Yeast culture is not growing, or growing slower than expected.

### Potential solution

We strongly recommend calculating the doubling time of the yeast strain being used for the experiments since doubling time can be influenced by the genetic background of the yeast as well as the media used for the experiments (rich versus synthetic). For wild type yeast (BY4741 strain), doubling times are ∼90 minutes in rich media, or ∼2 hours in synthetic media. If wild type BY4741 is not following this doubling time or not doubling at all, this would indicate a problem with yeast strain or media. To prevent these issues, we advise not using any yeast streaks that are older than 1 month. If there is any problem with yeast growth, we also suggest re-making the media with fresh components, purified water, and clean flasks rinsed with purified water.

### Problem 2

There is no powdered lysate after cryolysis.

### Potential solution

To prevent this, the cryolysis components (metal balls and capsules) should be fully dry. The cell scrape, and frozen buffer droplets should remain frozen prior to lysis.

### Problem 3

Baseline absorbance at 260 nm falls outside of bounds during Biocomp instrument set up.

### Potential solution

This issue may be caused by residual air or nucleic acid in the system. First, repeatedly purge the system with water from the rinse adaptor end, and from the system pump end. This should eliminate air. If the issue persists, perform a stringent wash with 0.2 M sodium hydroxide followed by 1% SDS and then with water.

### Problem 4

There is a smear in the size selection gel instead of the expected banding pattern observed in Figure 8.

### Potential solution

This could indicate RNase contamination in the buffers, so we strongly recommend using RNase-free buffers and water, and conducting experiments with clean gloves, and on a clean bench frequently cleaned with 70% ethanol.

### Problem 5

At the reverse transcription step, cDNA band is not visible for either the marker or the samples on the gel.

### Potential solution

If this occurs, size selection and all steps onwards need to be repeated. If the reverse transcription doesn’t work again, we strongly recommend confirming the sequence of the oligos used for reverse transcription and making sure to have non-expired reagents.

### Problem 6

At the reverse transcription step, cDNA band is visible for the marker but not for the samples.

### Potential solution

It is possible that the cDNA band is not visible for the sample, despite working for the marker. This could indicate the yield may be too low to see on the gel, but the library PCR may still work with the sample. If there is no band in the library PCR even with 13 cycles, then we recommend increasing the amount of input material at size selection step and repeating all the steps onwards.

## Resource availability

### Lead contact

Further information and requests for resources and reagents should be directed to and will be fulfilled by the lead contact, Sezen Meydan (sezen.meydan@vanderbilt.edu).

### Technical contact

Technical questions on executing this protocol should be directed to and will be answered by the technical contact, Sezen Meydan (sezen.meydan@vanderbilt.edu).

### Materials availability

This study did not generate novel reagents.

### Data and code availability

The gel images used for the figures are deposited in the Mendeley Data: https://doi.org/10.17632/8pc7wnctg6.1.

## Acknowledgments

We thank Nicholas Guydosh and Gustavo Silva for helpful discussions during the development of the protocol. The graphical abstract is created in BioRender. This work is supported by NIH 4R00GM144688-02 to S.M.

## Author contributions

P.H.A.-G., J.M., and S.M. performed experiments. P.H.A.-G., J.M., and S.M. wrote the manuscript. S.M. supervised the project.

## Declaration of interests

The authors declare no competing interests.
