# Supplementary material for: Protocol for Disome-seq to identify transcriptome-wide ribosome collisions in yeast cells
Source: STAR Protoc. 2025 Aug 26;6(3):104047. doi: 10.1016/j.xpro.2025.104047 (PMC12408389; doi:10.1016/j.xpro.2025.104047)
Supplement: Document S1. Table S1 [file mmc1.pdf]

Table S1. Oligonucleotides used in this protocol. Related to Key Resources Table.

**Name**	**Sequence (5' to 3')**	**Source**
**RNA size selection markers**		
25mer	rArUrGrUrArCrArCrGrGrArGrUrCrGrArGrCrArCrCrCrGrCrA	(McGlincy and Ingolia, 2017)[1]
34mer	rArUrGrUrArCrArCrGrGrArGrUrCrGrArGrCrArCrCrCrGrCrA rArCrGrCrGrArArUrG	
54mer	rArUrGrUrArCrArCrGrGrArGrUrCrGrArGrCrArCrCrCrGrCrA rArCrGrCrGrArArUrGrUrArCrArCrGrGrArGrUrCrGrArGrCrA rCrCrCrG	
68mer	rArUrGrUrArCrArCrGrGrArGrUrCrGrArGrCrArCrCrCrGrCrA rArCrGrCrGrArArUrGrUrArCrArCrGrGrArGrUrCrGrArGrCrA rCrCrCrGrCrArArCrGrCrGrArUrGrUrArCrA	
**Linker oligonucleotides**		
NI-810	5´-/5Phos/NNNNNATCGTAGATCGGAAGAGCACACGTCTGAA/3ddC/	(McGlincy and Ingolia, 2017)[1]
NI-811	5´-/5Phos/NNNNNAGCTAAGATCGGAAGAGCACACGTCTGAA/3ddC/	
NI-812	5´-/5Phos/NNNNNCGTAAAGATCGGAAGAGCACACGTCTGAA/3ddC/	
NI-813	5´-/5Phos/NNNNNCTAGAAGATCGGAAGAGCACACGTCTGAA/3ddC/	
NI-814	5´-/5Phos/NNNNNGATCAAGATCGGAAGAGCACACGTCTGAA/3ddC/	
NI-815	5´-/5Phos/NNNNNGCATAAGATCGGAAGAGCACACGTCTGAA/3ddC/	
**RT primer**		
NI-802	5´-/5Phos/NNAGATCGGAAGAGCGTCGTGTAGGGAAAGAG/iSp18/GTGACTGGAGTTCAGACGTGTGCTC	(McGlincy and Ingolia, 2017)[1]
**PCR primers**		
NI-NI-798	5´-AATGATACGGCGACCACCGAGATCTACACTCTTTCCCTACACGACGCTC	(McGlincy and Ingolia, 2017)[1]

NI-799	5´-CAAGCAGAAGACGGCATACGAGATCGTGATGTGACTGGAGTTCAGACGTGTG	
NI-822	5’-CAAGCAGAAGACGGCATACGAGATACATCGGTGACTGGAGTTCAGACGTGTG	
NI-823	5’-CAAGCAGAAGACGGCATACGAGATGCCTAAGTGACTGGAGTTCAGACGTGTG	
NI-824	5’-CAAGCAGAAGACGGCATACGAGATTGGTCAGTGACTGGAGTTCAGACGTGTG	
NI-825	5’-CAAGCAGAAGACGGCATACGAGATCACTGTGTGACTGGAGTTCAGACGTGTG	
